# Supplementary material for: Syndecans and glycosaminoglycans influence B-cell development and activation
Source: EMBO Rep. 2025 Mar 28;26(9):2435–58. doi: 10.1038/s44319-025-00432-6 (PMC12069707; doi:10.1038/s44319-025-00432-6)
Supplement: Supplementary file 8 — Expanded View Figures [file 44319_2025_432_MOESM8_ESM.pdf]

Expanded View Figures

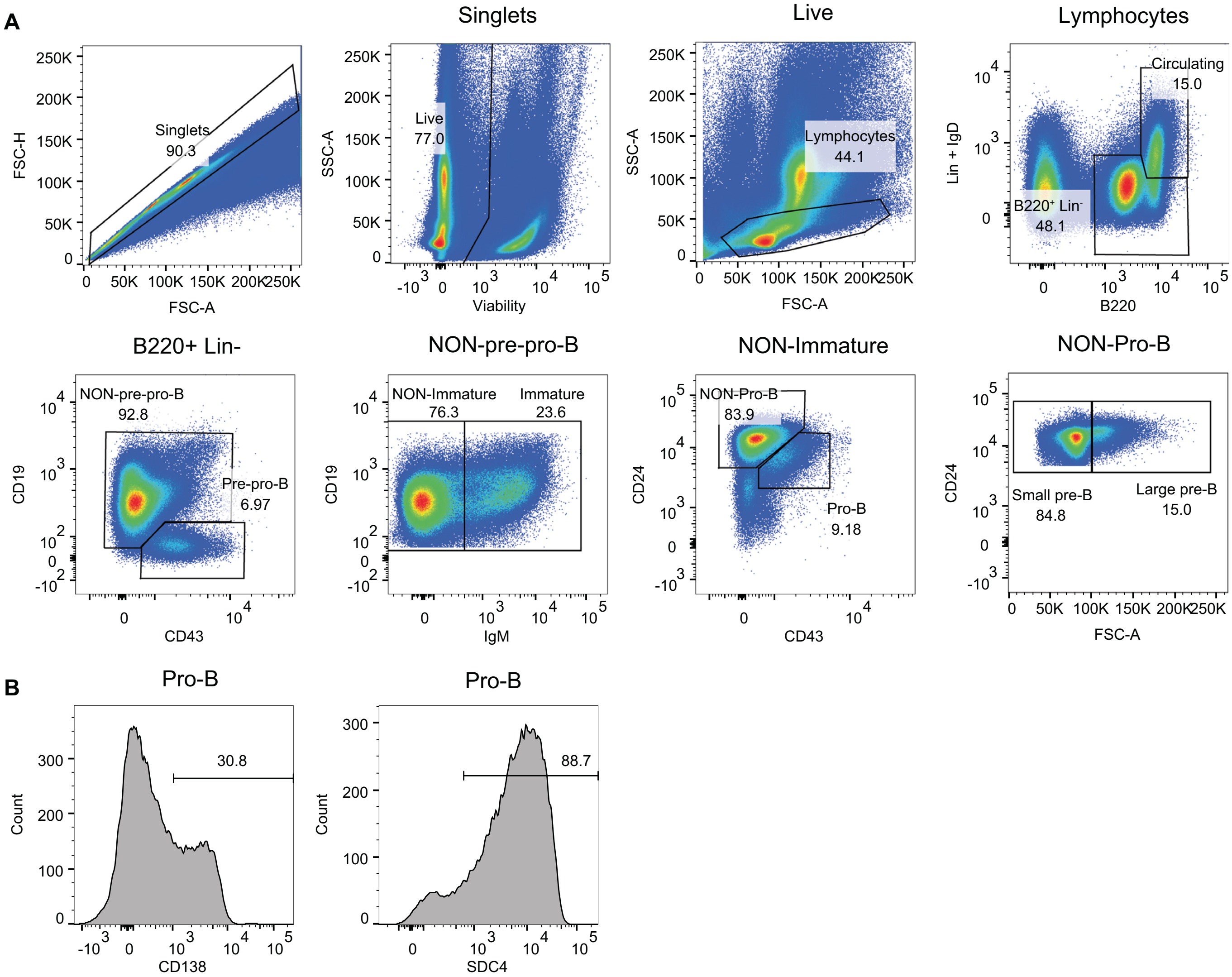

Figure EV1. Flow cytometric gating strategy for the identification of B-cell subsets undergoing maturation in the BM.

(**A**) Representative flow cytometric plots for the identification of the indicated stages of B-cell development in BM. Lin (Mac-1, Gr-1, CD3). (**B**) Representative histograms of CD138 and SDC4 expression on pro-B cells.

 © The Author(s)

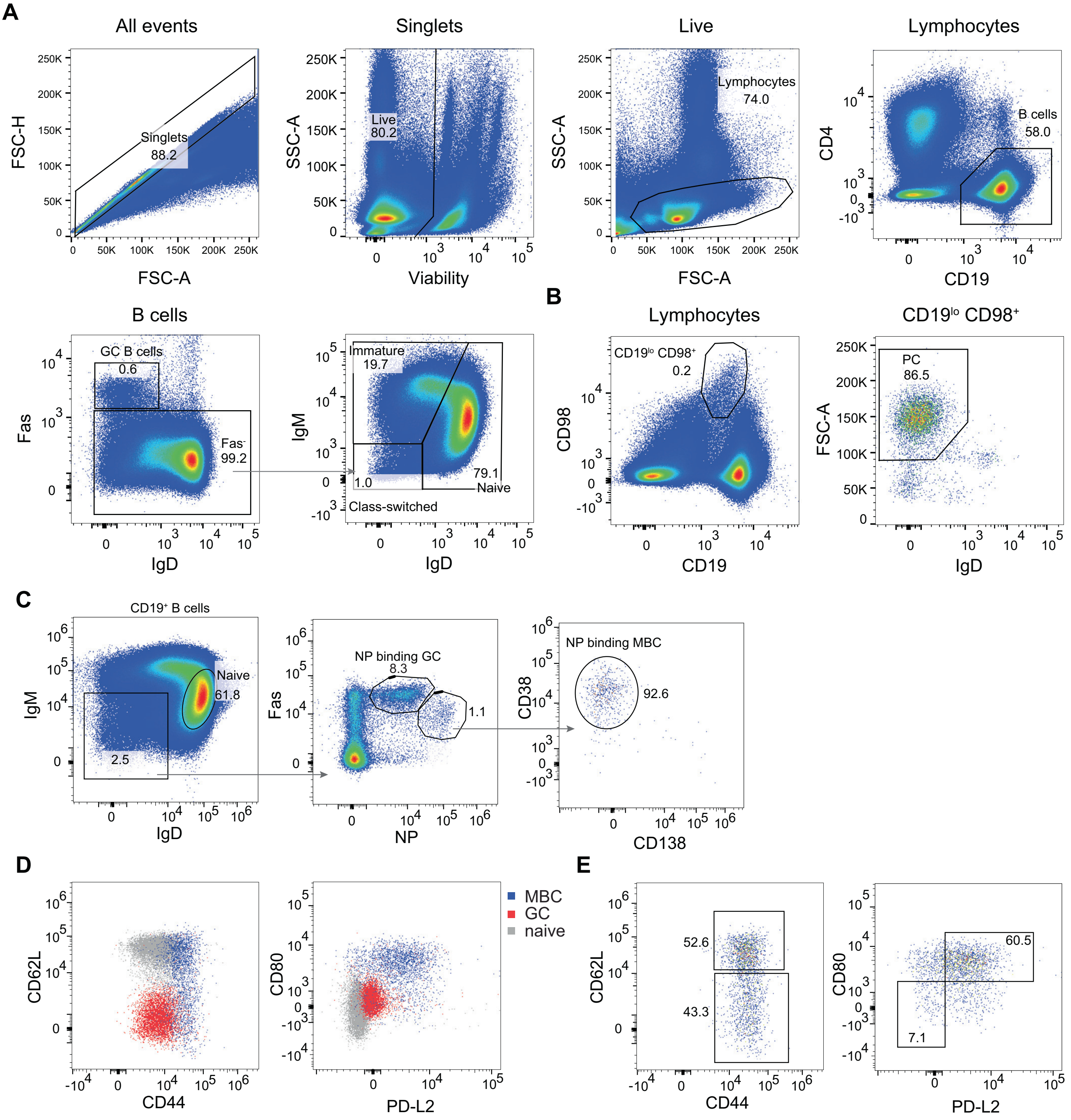

Figure EV2. Flow cytometric gating strategy for the identification of mature B cell subsets in the spleen.

(**A**, **B**) Representative flow cytometric plots for the identification of (**A**) B cells and B-cell subsets and (**B**) PC. (**C–E**) Analysis of antigen (NP)-specific germinal center (GC) and MBC 3 weeks post NP-KLH immunization. (**C**) MBC gating strategy, (**D**) color-coded overlay of indicated population showing expression of cell surface molecules with MBC subsets. (**E**) MBC subset gating.

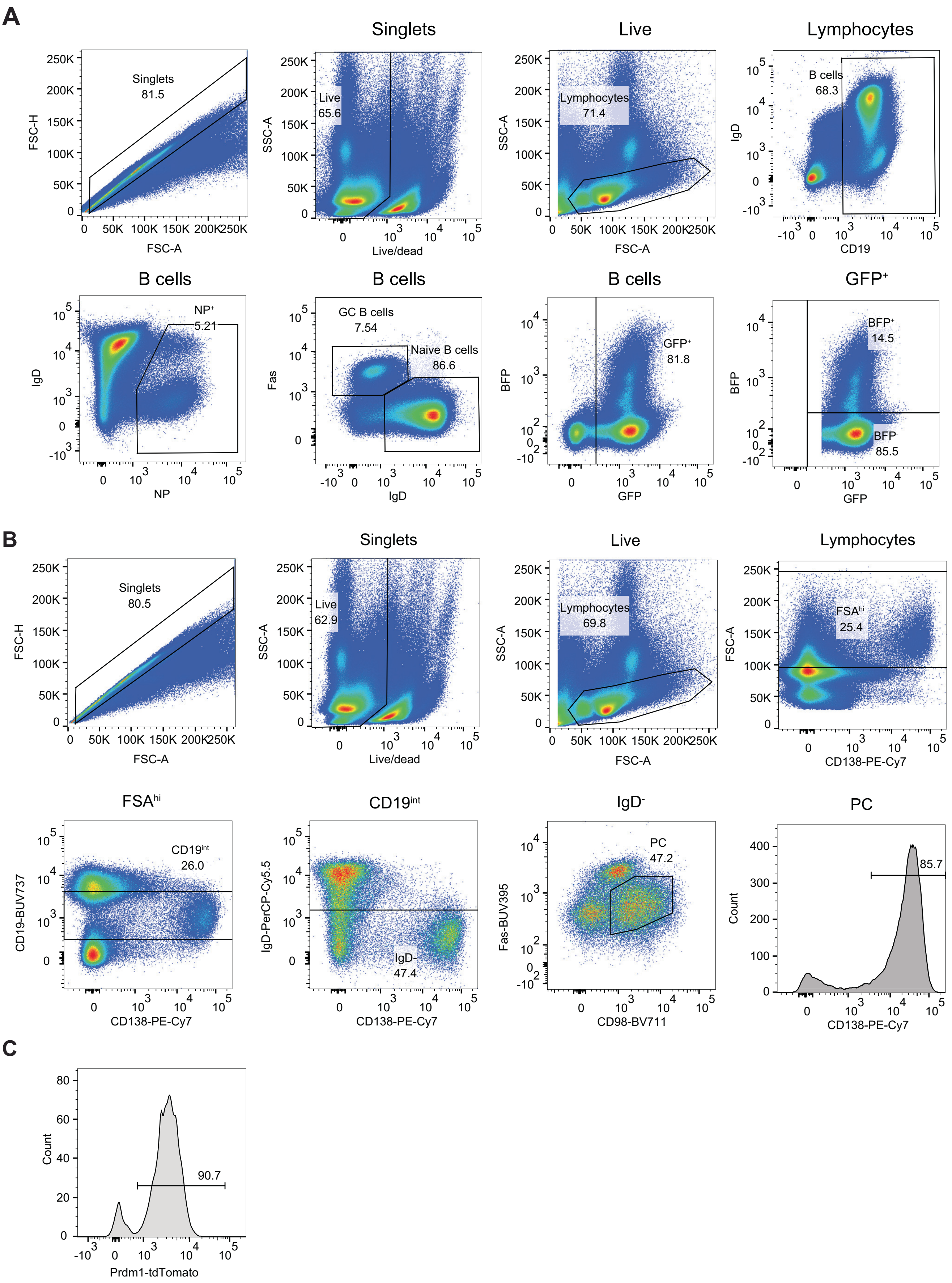
A
Singlets
Live
Lymphocytes
Singlets
81.5
FSC-H
FSC-A
Live
65.6
SSC-A
Live/dead
Lymphocytes
71.4
SSC-A
FSC-A
B cells
68.3
IgD
CD19
B cells
B cells
B cells
GFP⁺
NP⁺
5.21
IgD
NP
GC B cells
7.54
Naive B cells
86.6
Fas
IgD
GFP⁺
81.8
BFP
GFP
BFP⁺
14.5
BFP⁻
85.5
BFP
GFP
B
Singlets
Live
Lymphocytes
Singlets
80.5
FSC-H
FSC-A
Live
62.9
SSC-A
Live/dead
Lymphocytes
69.8
SSC-A
FSC-A
FSA^hi
25.4
FSC-A
CD138-PE-Cy7
FSA^hi
CD19^int
IgD⁻
PC
CD19^int
26.0
CD19-BUV737
CD138-PE-Cy7
IgD-
47.4
IgD-PerCP-Cy5.5
CD138-PE-Cy7
PC
47.2
Fas-BUV395
CD98-BV711
85.7
Count
CD138-PE-Cy7
C
90.7
Count
Prdm1-tdTomato

Figure EV3. Flow cytometric gating strategy for the identification of B-cell subsets from the spleen of reconstituted mice.

(**A**) Representative flow cytometry plots showing sequential gating steps to identify mature B-cell subsets and NP-specific B cells in the spleen of mice reconstituted with lentivirally transduced BM for CRISPR–Cas9 genome editing. (**B**) Representative flow cytometry plots for the identification of PC from the spleen of reconstituted mice. (**C**) CD138 independent PC gating strategy applied to splenocytes from mice expressing tdTomato under the control of the *Prdm1* locus.

© The Author(s)

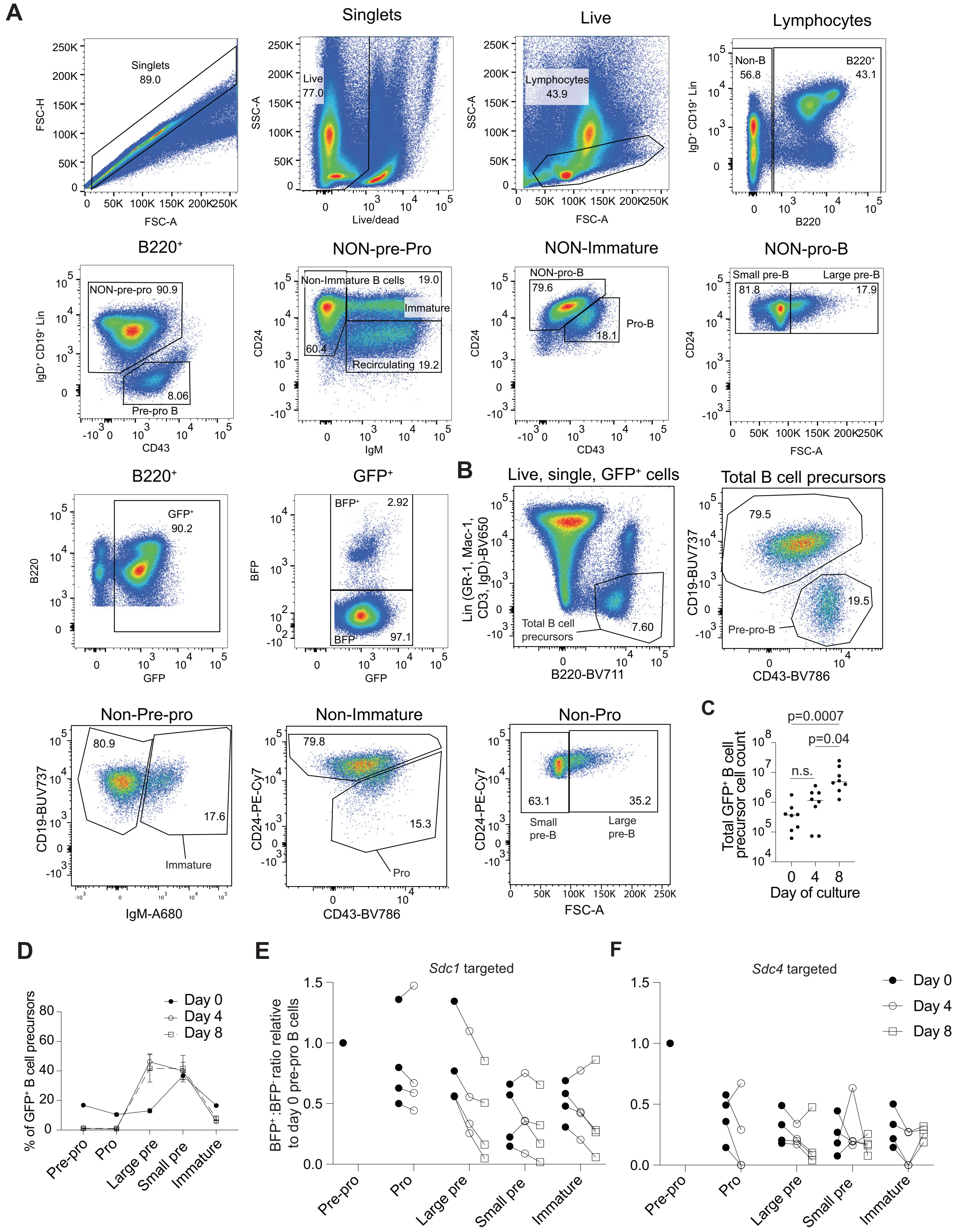
A
Singlets
Live
Lymphocytes
Singlets 89.0
Live 77.0
Lymphocytes 43.9
Non-B 56.8
B220⁺ 43.1
FSC-H
FSC-A
SSC-A
Live/dead
IgD⁺ CD19⁺ Lin
B220
B220⁺
NON-pre-Pro
NON-Immature
NON-pro-B
NON-pre-pro 90.9
Pre-pro B 8.06
Non-Immature B cells 19.0
Immature
60.4
Recirculating 19.2
NON-pro-B 79.6
Pro-B 18.1
Small pre-B 81.8
Large pre-B 17.9
CD43
IgM
CD24
GFP⁺
GFP⁺ 90.2
BFP⁺ 2.92
BFP⁻ 97.1
GFP
BFP
B
Live, single, GFP⁺ cells
Total B cell precursors
Lin (GR-1, Mac-1, CD3, IgD)-BV650
Total B cell precursors 7.60
B220-BV711
CD19-BUV737
79.5
19.5
Pre-pro-B
CD43-BV786
Non-Pre-pro
Non-Immature
Non-Pro
80.9
17.6
Immature
IgM-A680
79.8
15.3
Pro
CD24-PE-Cy7
63.1
35.2
Small pre-B
Large pre-B
C
p=0.0007
p=0.04
n.s.
Total GFP⁺ B cell precursor cell count
Day of culture
D
% of GFP⁺ B cell precursors
Day 0
Day 4
Day 8
Pre-pro
Pro
Large pre
Small pre
Immature
E
Sdc1 targeted
BFP⁺:BFP⁻ ratio relative to day 0 pre-pro B cells
F
Sdc4 targeted

 © The Author(s)

Figure EV4. Flow cytometric gating strategy for the identification of developing B cell subsets in the BM of mice reconstituted with lentiviral transduced Cas9 transgenic BM.

(**A**) Representative flow cytometry plots for the identification of the indicated stages of B-cell development in BM. Lin (Mac-1, Gr-1, CD3). (**B–F**) In vitro IL-7 mediated B-cell differentiation. gating strategy (**B**), total B-cell precursor cell lumbers ($GFP^{+}B220^{+}$ Lin^{-} cells) (**C**) and precursor subset composition (**D**) over time. (**E**) Proportional representation of *Sdc1* gene-targeted ($GFP^{+}BFP^{+}$) cell among $CAS9^{tg}$ BM-derived (GFP^{+}) B-cell precursors relative to Pre-pro-B cells at the start of culture (day 0). (**F**) Analysis as in (**E**) but for Sdc4-targeted cells. Statistical analysis in (**C**) by Kruskal–Wallis test with Dunn's multiple comparisons test. Data in (**D–F**) are pooled from two experiments. (**D**) shows means ($n = 8$) and SEM. Lines in (**E**) and (**F**) connect individual mice ($n = 4$). Some data points in pre-pro and pro-B cells are absent due to very low cell numbers at the respective time points. Source data are available online for this figure.

© The Author(s)

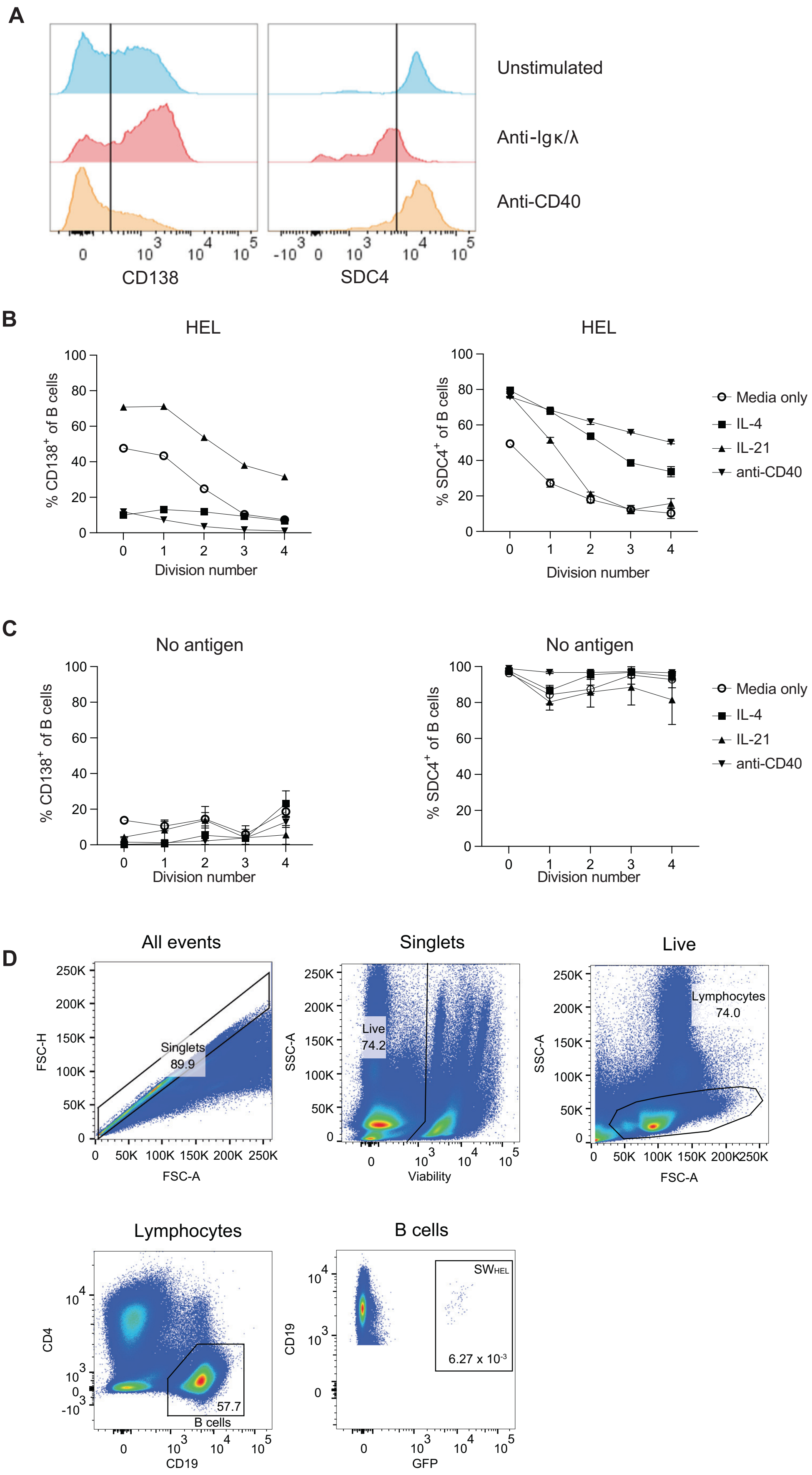
A
Unstimulated
Anti-Igκ/λ
Anti-CD40
CD138
SDC4
B
HEL
% CD138+ of B cells
% SDC4+ of B cells
Division number
Media only
IL-4
IL-21
anti-CD40
C
No antigen
% CD138+ of B cells
% SDC4+ of B cells
Division number
Media only
IL-4
IL-21
anti-CD40
D
All events
FSC-H
FSC-A
Singlets
89.9
Singlets
SSC-A
Viability
Live
74.2
Live
SSC-A
FSC-A
Lymphocytes
74.0
Lymphocytes
CD4
CD19
57.7
B cells
B cells
CD19
GFP
SWHEL
6.27 x 10-3

Figure EV5. Expression of CD138 and SDC4 on BCR-stimulated B cells decreases with consecutive divisions.

(**A**) CD138 and SDC4 expression on B cells stimulated for 3 days in culture with anti-Igκ and anti-Igλ or anti-CD40 antibody. (**B**, **C**) Expression of CD138 and SDC4 on RAG-$1^{-/-}$ SW_{HEL} B cells stimulated with (**B**) or without (**C**), HEL and IL-21, IL-4 or anti-CD40 (additional data for Fig. 3) showing mean and SD of technical triplicates, representative of 2 experiments. (**D**) Gating strategy for the identification of RAG-$1^{-/-}$ SW_{HEL} B cells 3 days post-transfer in the spleen of HEL^{WT}-OVA_{pep} immunized wild-type recipients. Source data are available online for this figure.

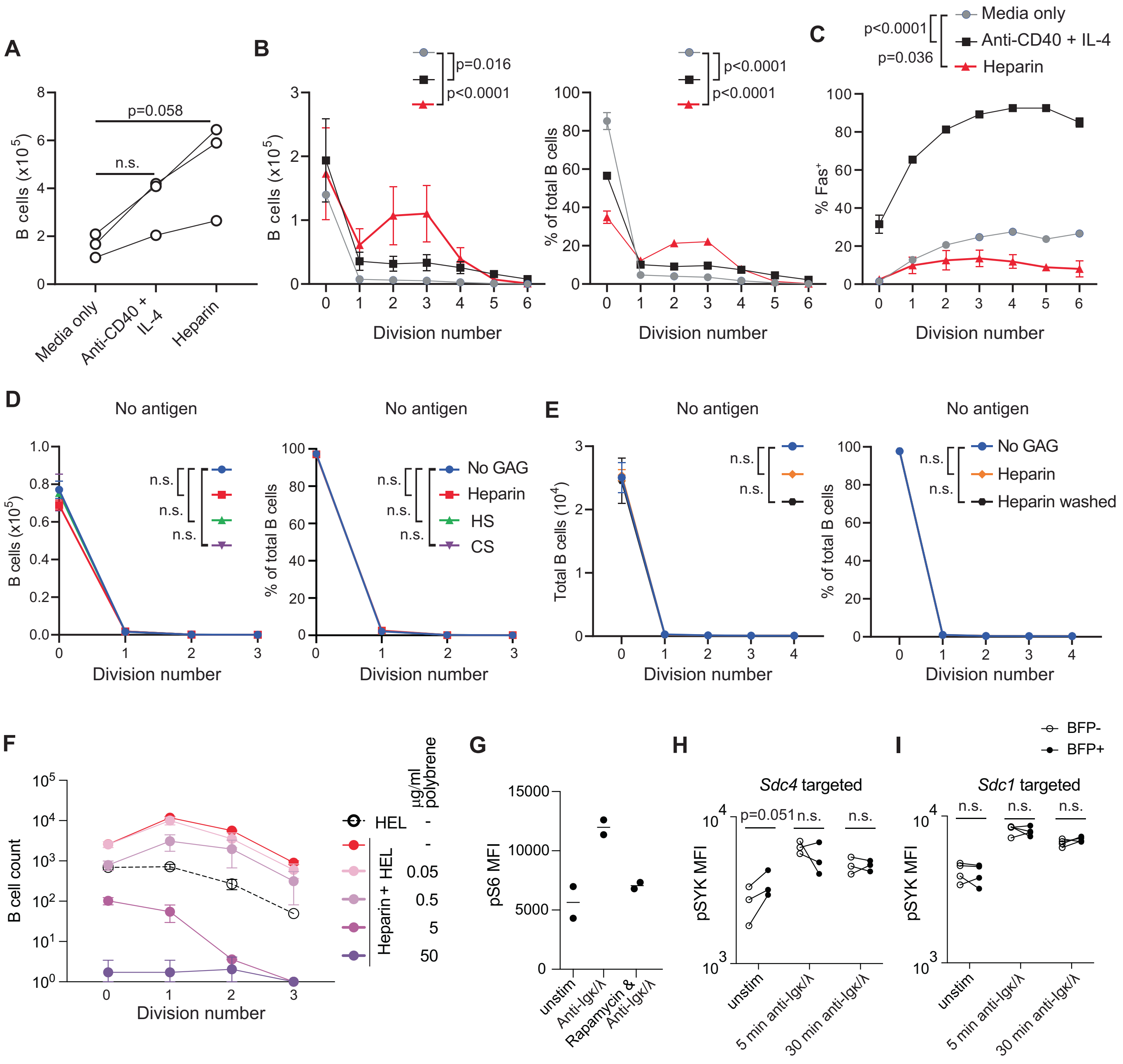

Figure EV6. Heparin at a high concentration induces B-cell proliferation without FAS upregulation.

(**A**) Total number of WT B cells, (**B**) number and proportion of B cells per division and (**C**) proportion of FAS^{+} B cells per division after 3 days of culture with high-dose heparin (250 IU/mL) or anti-CD40 and IL-4. (**D, E**) Number and proportion of RAG-1$^{-/-}$ SW_{HEL} B cells per division after 3 days of culture without HEL antigen and (**D**) with heparin (2.5 IU/mL), HS and CS or (**E**) after short-term pre-incubation with heparin (2.5 IU/mL) followed by washing and re-culture for 3 days (additional data for Fig. 4). (**F**) RAG-1$^{-/-}$ SW_{HEL} B cells count per CTV division peak after 3 days cell culture in the presence of HEL or HEL + heparin (2.5 IU/mL) with or without polybrene. (**G**) WT B cells ($n = 2$) were incubated for 30 min with anti-Igκ and anti-Igλ in the presence or absence of rapamycin and S6 phosphorylation (pS6) analyzed by flow cytometry. (**H, I**) Syk phosphorylation (pSyk) in B cells from BM-reconstituted mice (as shown in Fig. 3) comparing non-targeted to *Sdc4* (**H**) or *Sdc1* (**I**) sgRNA-targeted B cells. (**A–E**) are representative of 2 experiments and show mean of technical triplicates ($n = 3$) with error bars indicating standard deviation. Data in F are from one experiment, assayed in duplicates showing median and range. H and I show data from 3–4 mice (cells from each mouse were assayed in duplicates and means are shown) and statistical analysis by paired t test. Source data are available online for this figure.

 © The Author(s)
